# *trans*-Dichlorido­tetra­kis­[(di­methyl­phosphor­yl)methanaminium-κ*O*]cobalt(II) tetra­chloridocobaltate(II)

**DOI:** 10.1107/S1600536813008945

**Published:** 2013-04-10

**Authors:** Guido J. Reiss

**Affiliations:** a Institut für Anorganische Chemie und Strukturchemie, Lehrstuhl II: Material- und Strukturforschung, Heinrich-Heine-Universität Düsseldorf, Universitätsstrasse 1, D-40225 Düsseldorf, Germany

## Abstract

The asymmetric unit of the title structure, [CoCl_2_(C_3_H_11_NOP)_4_][CoCl_4_]_2_, consists of one half of the *trans*-dichlorido­tetra­kis­[(di­methyl­phosphor­yl)methanaminium]cobalt(II) tetra­cation lying on an inversion center and one tetra­chloridocobaltate(II) dianion on a general position. Four *O*-coordinated cationic (di­methyl­phosphor­yl)methanaminium (dpmaH^+^) ligands occupy the equatorial coordination sites, whereas the chloride ligands occupy axial positions of the roughly o­cta­hedral coordination polyhedron of the cobalt metal center. Intra­molecular hydrogen bonds between the aminium groups and the O atom of the phosphoryl groups and additional hydrogen bonds between the aminium groups and the chloride ligands are present. Furthermore, four of the six H atoms not involved in intra­molecular bonding of each cobalt(II) tetra­cation form weak hydrogen bonds to four adjacent tetra­chloridocobaltate(II) counter-anions. By these inter­molecular hydrogen bonds, one-dimensional polymeric strands are formed along the *b*-axis direction. The hydrogen bonding is analyzed using the graph-set method and the structural similarity with dpmaHCl is discussed.

## Related literature

For related dpma compounds, see: Dodoff *et al.* (1990 ▶); Borisov *et al.* (1994 ▶); Trendafilova *et al.* (1997 ▶); Kochel (2009 ▶); Reiss & Jörgens (2012 ▶); van Megen *et al.* (2013 ▶). For a definition of the term tecton, see: Brunet *et al.* (1997 ▶). For related methyl­phosphinic acids and their derivatives, see: Reiss & Engel (2008 ▶); Meyer *et al.* (2010 ▶). For graph-set theory and its applications, see: Etter *et al.* (1990 ▶); Bernstein *et al.* (1995 ▶); Grell *et al.* (2002 ▶). For related cobalt complexes, see: Kubíčk *et al.* (2003 ▶); Girma *et al.* (2005 ▶); Guzei *et al.* (2010 ▶).
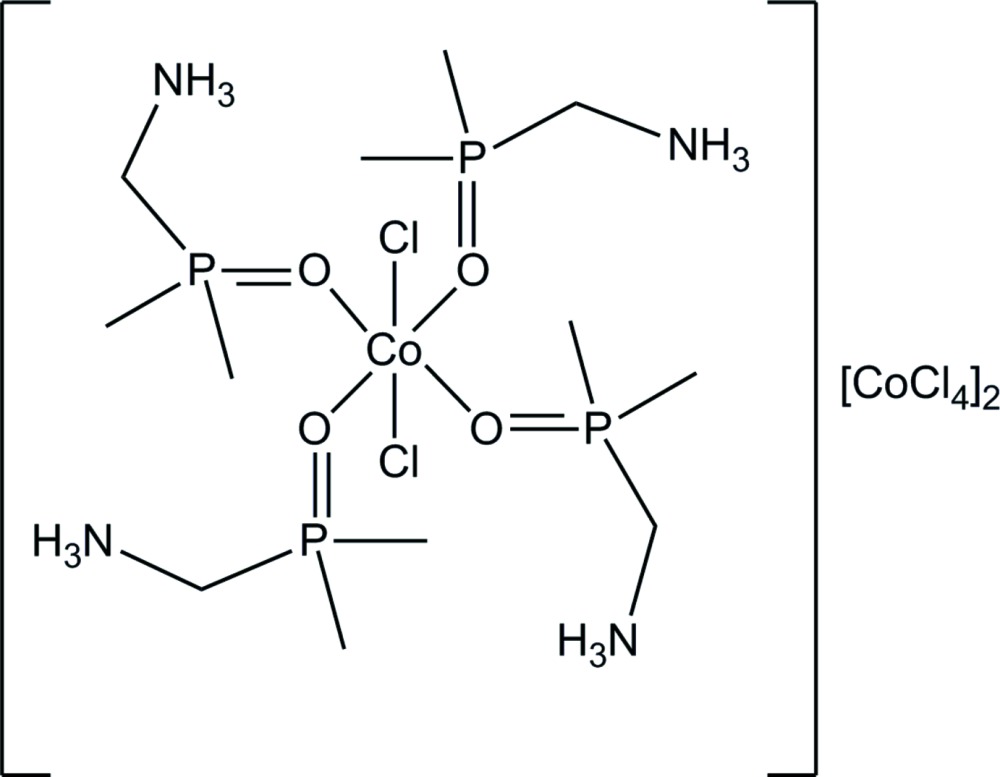


## Experimental

### 

#### Crystal data

[CoCl_2_(C_3_H_11_NOP)_4_][CoCl_4_]_2_
*M* *_r_* = 963.68
Triclinic, 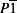

*a* = 7.7748 (3) Å
*b* = 11.1557 (5) Å
*c* = 12.1205 (5) Å
α = 110.738 (4)°
β = 97.688 (4)°
γ = 104.331 (5)°
*V* = 923.66 (8) Å^3^
*Z* = 1
Mo *K*α radiation
μ = 2.25 mm^−1^
*T* = 173 K
0.76 × 0.33 × 0.08 mm

#### Data collection

Oxford Xcalibur diffractometer
Absorption correction: analytical [*CrysAlis PRO* (Oxford Diffraction, 2009 ▶), based on expressions derived by Clark & Reid (1995 ▶)] *T*_min_ = 0.402, *T*_max_ = 0.838
15687 measured reflections
4920 independent reflections
4552 reflections with *I* > 2σ(*I*)
*R*_int_ = 0.020

#### Refinement

*R*[*F* ^2^ > 2σ(*F* ^2^)] = 0.018
*wR*(*F* ^2^) = 0.042
*S* = 1.09
4920 reflections
197 parameters
H atoms treated by a mixture of independent and constrained refinement
Δρ_max_ = 0.49 e Å^−3^
Δρ_min_ = −0.38 e Å^−3^

### 

Data collection: *CrysAlis PRO* (Oxford Diffraction, 2009 ▶); cell refinement: *CrysAlis PRO*; data reduction: *CrysAlis PRO*; program(s) used to solve structure: *SHELXS97* (Sheldrick, 2008 ▶); program(s) used to refine structure: *SHELXL2013* (Sheldrick, 2008 ▶); molecular graphics: *DIAMOND* (Brandenburg, 2012 ▶); software used to prepare material for publication: *publCIF* (Westrip, 2010 ▶).

## Supplementary Material

Crystal structure: contains datablock(s) I, global. DOI: 10.1107/S1600536813008945/sj5313sup1.cif

Structure factors: contains datablock(s) I. DOI: 10.1107/S1600536813008945/sj5313Isup2.hkl

Additional supplementary materials:  crystallographic information; 3D view; checkCIF report

## Figures and Tables

**Table 1:** Hydrogen-bond geometry (Å, °)

*D*—H⋯*A*	*D*—H	H⋯*A*	*D*⋯*A*	*D*—H⋯*A*
N1—H11⋯Cl1	0.867 (19)	2.437 (19)	3.1879 (12)	145.3 (16)
N1—H11⋯Cl24^i^	0.867 (19)	2.730 (19)	3.2573 (13)	120.5 (15)
N1—H12⋯O2^ii^	0.88 (2)	2.16 (2)	2.9504 (16)	150.3 (17)
N1—H13⋯Cl23^ii^	0.87 (2)	2.38 (2)	3.2403 (12)	171.3 (17)
N2—H22⋯Cl1	0.920 (19)	2.250 (19)	3.1697 (13)	177.8 (15)
N2—H21⋯Cl21^iii^	0.924 (19)	2.288 (19)	3.2124 (12)	177.8 (16)
N2—H23⋯Cl22^i^	0.86 (2)	2.71 (2)	3.3612 (13)	134.0 (16)
N2—H23⋯Cl24^i^	0.86 (2)	2.70 (2)	3.2989 (12)	128.1 (16)

Symmetry codes: (i) 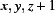; (ii) 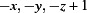; (iii) 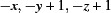.

**Fig. 1.**
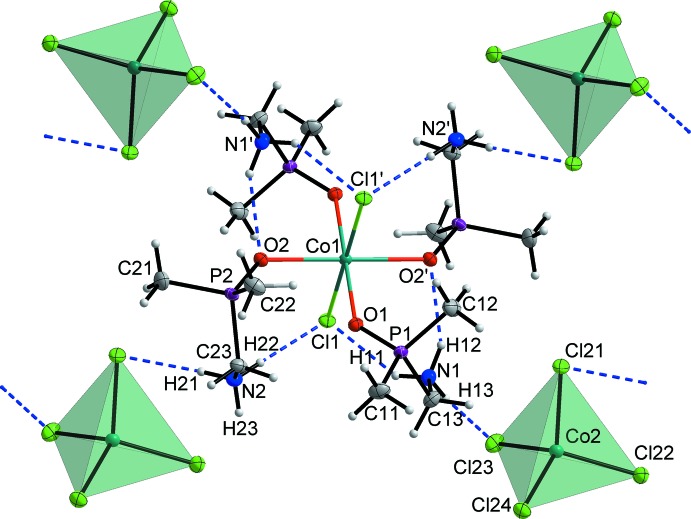
The title structure consisting of [CoCl2(dpmaH)4] cations which form hydrogen bonds to neighbouring [CoCl4]- anions is shown (ellipsoids are drawn at the 50% probability level, the asymmetric unit is labeled).

**Fig. 2.**
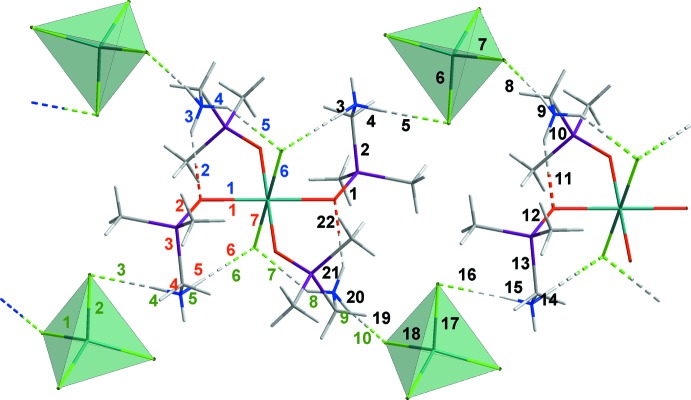
Wireframe sketch of the title structure showing the basic graph-sets (blue numbers: S22(6), red numbers: S11(7), green numbers: C34(10), black numbers: R66(22)).

**Fig. 3.**
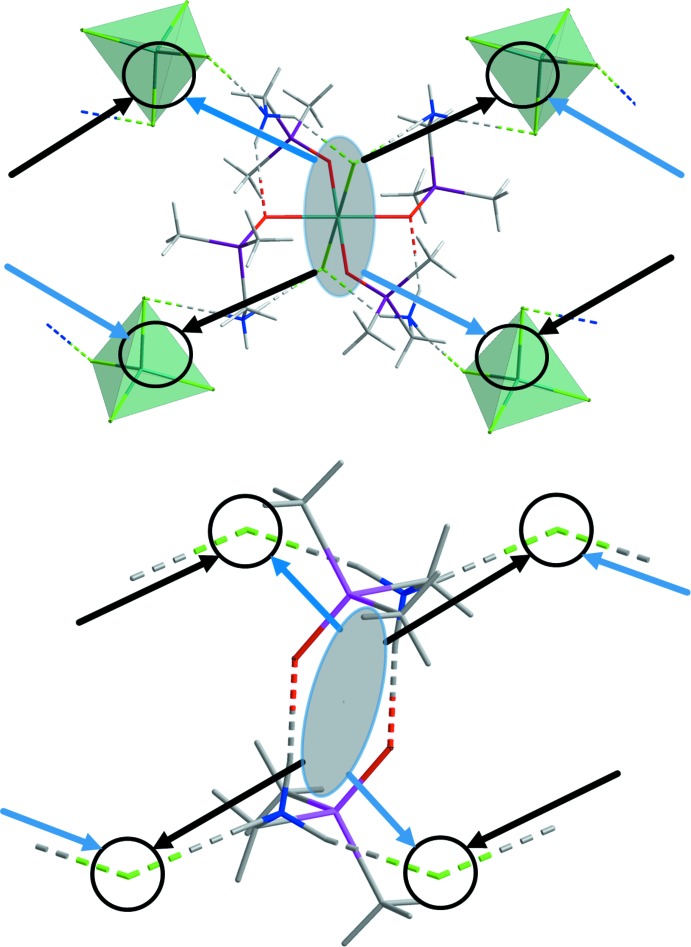
Constructor-graphs of the title structure and of dpmaHCl are shown in the context of their real structures to illustrate the structural similarity.

## supplementary crystallographic information

### Comment

It is well known that the dpma ligand (dpma = (dimethylphosphoryl)methanamine) is able to coordinate a variety of transition metals (Kochel, 2009; Trendafilova *et al.*, 1997; Borisov *et al.*, 1994; Dodoff *et al.*, 1990.). Recently, it has been shown that the mono-protonated dpmaH^+^ cation is a potent tecton (for the term tecton, see Brunet *et al.*, 1997) to construct hydrogen bonded polymeric structures (Reiss & Jörgens, 2012; van Megen *et al.*, 2013). This study is part of our continuing interest in the construction of hydrogen bonded architectures using tectons based on phosphinic acid derivatives (Reiss & Engel, 2008; Meyer *et al.*, 2010).

The asymmetric unit of the title structure consists of one half of a fourfold charged *trans*-dichloridotetrakis((dimethylphosphoryl)methanaminium)cobalt(II) complex located on a center of inversion and a tetrachloridocobaltate(II) dianion at a general position. In the complex cation the four *O*-coordinated dpmaH^+^ ligands occupy the equatorial coordination sites, whereas the chlorido ligands occupy axial positions in this roughly octahedral complex cation. The Co—O and Co—Cl distances are in the expected ranges (Girma *et al.*, 2005, Guzei *et al.*, 2010). The same applies to the geometrical parameters of the two crystallographically independent dpmaH ligands which are very similar and are in accord with the dpmaH^+^ cation in dpmaHCl (Reiss & Jörgens, 2012). Each chlorido ligand of the cationic complex accepts two intramolecular hydrogen bonds of two neighbouring aminium groups (N1 and N2). There is at least one more example of the occurrence of such an intramolecular hydrogen bond between a chlorido ligand and the aminium group of a coordinated ligand at the same metal center (Kubíčk *et al.*, 2003). Furthermore, intramolecular hydrogen bonding occurs between two crystallographically dependent aminium groups (N1, N1') each donating a hydrogen bond to the O-atoms (O2 and O2') of the two other dpmaH^+^ ligands (Table 1, Fig. 1). Significantly different Co–O bond lengths (Co–O1 = 2.0738 (9) Å and Co–O2 = 2.1673 (9) Å) may be caused by this hydrogen bonding situation. Four of the six hydrogen atoms of aminium groups of each cationic complex, which are not involved in intramolecular hydrogen bonds, form hydrogen bonds to four adjacent tetrachloridocobaltate(II) dianions (Fig. 1). The tetrachloridocobaltate(II) dianion shows a seriously distorted tetrahedral geometry with Co–Cl distances from 2.2487 (4) Å to 2.3024 (4) Å and angles between 104.23 (1)° to 119.35 (1)°. Also for this ion the longest Co–Cl distances are associated with the chlorido ligands involved in hydrogen bonds. Cationic and anionic tectons construct a one-dimensional, hydrogen-bonded polymer along the *b* direction. The hydrogen bonding motifs can be classified using graph-set descriptors (Etter *et al.*, 1990, Bernstein *et al.*, 1995) as S^2^_2_(6) and S^1^_1_(7) for the intramolecular rings and as *C*^3^_4_(10) for the backbone connection along the strands (Fig. 2). A third level graph-set is found (*R*^6^_6_(22)) for the rings formed within the strands (Fig. 2). As these graph-sets seem to be unique to this class of compounds they alone are of limited value for a comparison with related structures. A better method to work out the key features of a structure is the use of the so-called constructor-graph representation (Grell *et al.*, 2002). In this case, the complex cation can be reduced to a tecton that is able to donate at least four hydrogen bonds and the tetrachloridocobalte to a tecton that accepts at least two hydrogen bonds. Thus, the close relation of the title structure with the structure of dpmaHCl is inevitably clear (Fig. 3).

### Experimental

For the synthesis of the title compound, equimolar amounts of dpma and cobalt(II)chloride tetrahydrate were dissolved in concentrated hydrochloric acid. Slow evaporation of this solution at room temperature yielded crystals suitable for a crystallographic structure determination.

### Refinement

H atoms at the methyl groups were identified in difference syntheses, idealized and refined using rigid groups allowed to rotate about the P—C bond (AFIX 137 option of the *SHELXL* program; *U*_iso_(H) = 1.5*U*_eq_(C)) H-Atom at the methylene group using a riding model (AFIX 23 option of the *SHELXL* program; *U*_iso_(H) = 1.2*U*_eq_(C)). The coordinates of hydrogen atoms at the aminium groups were refined unrestrictedly with individual *U*_iso_values.

### Crystal data

**Table:**

[CoCl_2_(C_3_H_11_NOP)_4_][CoCl_4_]_2_	*Z* = 1
*M**_r_* = 963.68	*F*(000) = 487
Triclinic, *P*1	*D*_x_ = 1.732 Mg m^−^^3^
Hall symbol: -P 1	Mo *K*α radiation, λ = 0.71073 Å
*a* = 7.7748 (3) Å	Cell parameters from 13303 reflections
*b* = 11.1557 (5) Å	θ = 2.9–32.6°
*c* = 12.1205 (5) Å	µ = 2.25 mm^−^^1^
α = 110.738 (4)°	*T* = 173 K
β = 97.688 (4)°	Block, blue
γ = 104.331 (5)°	0.76 × 0.33 × 0.08 mm
*V* = 923.66 (8) Å^3^	

### Data collection

**Table:**

Oxford Xcalibur diffractometer	4920 independent reflections
Graphite monochromator	4552 reflections with *I* > 2σ(*I*)
Detector resolution: 16.2711 pixels mm^-1^	*R*_int_ = 0.020
ω scans	θ_max_ = 29.0°, θ_min_ = 2.9°
Absorption correction: analytical [*CrysAlis PRO* (Oxford Diffraction, 2009), based on expressions derived by Clark & Reid (1995)]	*h* = −10→10
*T*_min_ = 0.402, *T*_max_ = 0.838	*k* = −15→15
15687 measured reflections	*l* = −16→16

### Refinement

**Table:**

Refinement on *F*^2^	Primary atom site location: structure-invariant direct methods
Least-squares matrix: full	Secondary atom site location: difference Fourier map
*R*[*F*^2^ > 2σ(*F*^2^)] = 0.018	Hydrogen site location: difference Fourier map
*wR*(*F*^2^) = 0.042	H atoms treated by a mixture of independent and constrained refinement
*S* = 1.09	*w* = 1/[σ^2^(*F*_o_^2^) + (0.012*P*)^2^ + 0.5*P*], where *P* = (*F*_o_^2^ + 2*F*_c_^2^)/3
4920 reflections	(Δ/σ)_max_ = 0.001
197 parameters	Δρ_max_ = 0.49 e Å^−^^3^
0 restraints	Δρ_min_ = −0.38 e Å^−^^3^

### Special details

**Table:**

Experimental. CrysAlisPro, Oxford Diffraction Ltd., Version 1.171.34.44 Analytical numeric absorption correction using a multifaceted crystal model based on expressions derived by R.C. Clark & J.S. Reid. (Clark & Reid, 1995).
Geometry. All e.s.d.'s (except the e.s.d. in the dihedral angle between two l.s. planes) are estimated using the full covariance matrix. The cell e.s.d.'s are taken into account individually in the estimation of e.s.d.'s in distances, angles and torsion angles; correlations between e.s.d.'s in cell parameters are only used when they are defined by crystal symmetry. An approximate (isotropic) treatment of cell e.s.d.'s is used for estimating e.s.d.'s involving l.s. planes.

### Fractional atomic coordinates and isotropic or equivalent isotropic displacement parameters (Å^2^)

**Table:**

	*x*	*y*	*z*	*U*_iso_*/*U*_eq_	
Co1	0.0000	0.0000	0.5000	0.00707 (5)	
Cl1	−0.22848 (4)	0.05961 (3)	0.61151 (3)	0.01037 (6)	
P1	0.27356 (4)	−0.01270 (3)	0.73117 (3)	0.00824 (6)	
C11	0.48106 (18)	0.09673 (14)	0.84045 (13)	0.0149 (3)	
H11A	0.5714	0.1238	0.7997	0.022*	
H11B	0.5241	0.0503	0.8858	0.022*	
H11C	0.4599	0.1753	0.8949	0.022*	
C12	0.31916 (19)	−0.15805 (14)	0.63526 (13)	0.0149 (3)	
H12A	0.2069	−0.2229	0.5800	0.022*	
H12B	0.3725	−0.1973	0.6841	0.022*	
H12C	0.4027	−0.1323	0.5899	0.022*	
C13	0.11865 (17)	−0.06627 (14)	0.81714 (12)	0.0117 (2)	
H13A	0.1385	−0.1437	0.8293	0.014*	
H13B	0.1417	0.0064	0.8963	0.014*	
N1	−0.07340 (15)	−0.10285 (12)	0.74832 (11)	0.0112 (2)	
H11	−0.093 (2)	−0.0313 (19)	0.7437 (17)	0.022 (5)*	
H12	−0.094 (3)	−0.162 (2)	0.6731 (19)	0.026 (5)*	
H13	−0.149 (3)	−0.1352 (19)	0.7854 (18)	0.024 (5)*	
O1	0.18513 (12)	0.05528 (9)	0.66322 (8)	0.00960 (17)	
P2	0.25797 (4)	0.32277 (3)	0.61086 (3)	0.00895 (6)	
C21	0.2810 (2)	0.47433 (13)	0.58704 (13)	0.0158 (3)	
H21A	0.3016	0.4603	0.5077	0.024*	
H21B	0.3827	0.5461	0.6477	0.024*	
H21C	0.1710	0.4981	0.5929	0.024*	
C22	0.47331 (18)	0.29394 (14)	0.61459 (13)	0.0152 (3)	
H22A	0.4593	0.2039	0.6092	0.023*	
H22B	0.5591	0.3581	0.6894	0.023*	
H22C	0.5177	0.3043	0.5472	0.023*	
C23	0.22948 (18)	0.36089 (13)	0.76527 (12)	0.0119 (2)	
H23A	0.2637	0.2967	0.7933	0.014*	
H23B	0.3122	0.4504	0.8183	0.014*	
N2	0.03979 (16)	0.35581 (12)	0.77545 (11)	0.0122 (2)	
H21	0.003 (2)	0.4202 (18)	0.7562 (17)	0.019 (4)*	
H22	−0.041 (2)	0.2708 (19)	0.7275 (17)	0.018 (4)*	
H23	0.035 (3)	0.3711 (19)	0.8492 (19)	0.026 (5)*	
O2	0.10036 (12)	0.20618 (9)	0.51575 (8)	0.00961 (17)	
Co2	0.17901 (2)	0.34799 (2)	0.10713 (2)	0.01000 (4)	
Cl21	0.08194 (5)	0.41433 (3)	0.28405 (3)	0.01496 (7)	
Cl22	0.27225 (5)	0.53299 (3)	0.06796 (3)	0.01464 (7)	
Cl23	0.38534 (4)	0.24331 (3)	0.14315 (3)	0.01438 (7)	
Cl24	−0.04975 (4)	0.19017 (3)	−0.05026 (3)	0.01361 (6)	

### Atomic displacement parameters (Å^2^)

**Table:**

	*U*^11^	*U*^22^	*U*^33^	*U*^12^	*U*^13^	*U*^23^
Co1	0.00685 (11)	0.00721 (11)	0.00612 (11)	0.00138 (8)	0.00071 (9)	0.00240 (9)
Cl1	0.00962 (13)	0.01193 (13)	0.00975 (14)	0.00396 (11)	0.00322 (11)	0.00394 (11)
P1	0.00786 (14)	0.00935 (14)	0.00738 (15)	0.00274 (11)	0.00126 (11)	0.00342 (12)
C11	0.0116 (6)	0.0186 (7)	0.0110 (6)	0.0019 (5)	−0.0005 (5)	0.0053 (5)
C12	0.0174 (7)	0.0158 (6)	0.0134 (7)	0.0098 (5)	0.0037 (5)	0.0050 (5)
C13	0.0104 (6)	0.0144 (6)	0.0115 (6)	0.0030 (5)	0.0023 (5)	0.0073 (5)
N1	0.0106 (5)	0.0111 (5)	0.0128 (6)	0.0026 (4)	0.0036 (4)	0.0060 (5)
O1	0.0100 (4)	0.0099 (4)	0.0087 (4)	0.0029 (3)	0.0014 (3)	0.0040 (3)
P2	0.00928 (15)	0.00761 (14)	0.00711 (15)	0.00123 (11)	0.00126 (12)	0.00099 (12)
C21	0.0194 (7)	0.0099 (6)	0.0156 (7)	0.0016 (5)	0.0030 (5)	0.0048 (5)
C22	0.0098 (6)	0.0157 (6)	0.0147 (7)	0.0026 (5)	0.0017 (5)	0.0015 (5)
C23	0.0132 (6)	0.0131 (6)	0.0086 (6)	0.0059 (5)	0.0020 (5)	0.0027 (5)
N2	0.0149 (6)	0.0124 (5)	0.0101 (6)	0.0047 (4)	0.0046 (5)	0.0045 (5)
O2	0.0094 (4)	0.0082 (4)	0.0087 (4)	0.0008 (3)	0.0005 (3)	0.0024 (3)
Co2	0.01132 (8)	0.00894 (8)	0.00817 (8)	0.00225 (6)	0.00091 (7)	0.00285 (7)
Cl21	0.02275 (16)	0.01359 (14)	0.01120 (15)	0.00812 (12)	0.00686 (12)	0.00548 (12)
Cl22	0.01898 (16)	0.01075 (14)	0.01158 (15)	0.00080 (12)	0.00219 (12)	0.00476 (12)
Cl23	0.01173 (14)	0.01845 (15)	0.01554 (16)	0.00614 (12)	0.00327 (12)	0.00890 (13)
Cl24	0.01358 (14)	0.01152 (14)	0.01105 (15)	0.00125 (11)	−0.00131 (11)	0.00264 (12)

### Geometric parameters (Å, º)

**Table:**

Co1—O1^i^	2.0737 (9)	N1—H13	0.87 (2)
Co1—O1	2.0737 (9)	P2—O2	1.5144 (9)
Co1—O2^i^	2.1671 (9)	P2—C22	1.7791 (14)
Co1—O2	2.1671 (9)	P2—C21	1.7843 (14)
Co1—Cl1^i^	2.4525 (3)	P2—C23	1.8238 (14)
Co1—Cl1	2.4526 (3)	C21—H21A	0.9600
P1—O1	1.5083 (9)	C21—H21B	0.9600
P1—C11	1.7763 (14)	C21—H21C	0.9600
P1—C12	1.7794 (14)	C22—H22A	0.9600
P1—C13	1.8204 (13)	C22—H22B	0.9600
C11—H11A	0.9600	C22—H22C	0.9600
C11—H11B	0.9600	C23—N2	1.4852 (17)
C11—H11C	0.9600	C23—H23A	0.9700
C12—H12A	0.9600	C23—H23B	0.9700
C12—H12B	0.9600	N2—H21	0.924 (19)
C12—H12C	0.9600	N2—H22	0.920 (19)
C13—N1	1.4886 (17)	N2—H23	0.86 (2)
C13—H13A	0.9700	Co2—Cl22	2.2485 (4)
C13—H13B	0.9700	Co2—Cl24	2.2507 (4)
N1—H11	0.867 (19)	Co2—Cl23	2.2866 (4)
N1—H12	0.88 (2)	Co2—Cl21	2.3024 (4)
			
O1^i^—Co1—O1	180.00 (4)	C13—N1—H13	109.9 (13)
O1^i^—Co1—O2^i^	88.92 (3)	H11—N1—H13	109.5 (17)
O1—Co1—O2^i^	91.08 (3)	H12—N1—H13	109.9 (17)
O1^i^—Co1—O2	91.08 (3)	P1—O1—Co1	138.08 (6)
O1—Co1—O2	88.92 (3)	O2—P2—C22	113.83 (6)
O2^i^—Co1—O2	180.0	O2—P2—C21	111.21 (6)
O1^i^—Co1—Cl1^i^	89.89 (3)	C22—P2—C21	107.23 (7)
O1—Co1—Cl1^i^	90.11 (3)	O2—P2—C23	112.87 (6)
O2^i^—Co1—Cl1^i^	89.54 (3)	C22—P2—C23	104.79 (6)
O2—Co1—Cl1^i^	90.46 (3)	C21—P2—C23	106.37 (6)
O1^i^—Co1—Cl1	90.11 (3)	P2—C21—H21A	109.5
O1—Co1—Cl1	89.88 (3)	P2—C21—H21B	109.5
O2^i^—Co1—Cl1	90.46 (3)	H21A—C21—H21B	109.5
O2—Co1—Cl1	89.54 (3)	P2—C21—H21C	109.5
Cl1^i^—Co1—Cl1	180.0	H21A—C21—H21C	109.5
O1—P1—C11	113.52 (6)	H21B—C21—H21C	109.5
O1—P1—C12	113.89 (6)	P2—C22—H22A	109.5
C11—P1—C12	107.60 (7)	P2—C22—H22B	109.5
O1—P1—C13	108.03 (6)	H22A—C22—H22B	109.5
C11—P1—C13	105.63 (6)	P2—C22—H22C	109.5
C12—P1—C13	107.70 (7)	H22A—C22—H22C	109.5
P1—C11—H11A	109.5	H22B—C22—H22C	109.5
P1—C11—H11B	109.5	N2—C23—P2	113.44 (9)
H11A—C11—H11B	109.5	N2—C23—H23A	108.9
P1—C11—H11C	109.5	P2—C23—H23A	108.9
H11A—C11—H11C	109.5	N2—C23—H23B	108.9
H11B—C11—H11C	109.5	P2—C23—H23B	108.9
P1—C12—H12A	109.5	H23A—C23—H23B	107.7
P1—C12—H12B	109.5	C23—N2—H21	112.8 (11)
H12A—C12—H12B	109.5	C23—N2—H22	110.3 (11)
P1—C12—H12C	109.5	H21—N2—H22	110.3 (16)
H12A—C12—H12C	109.5	C23—N2—H23	108.5 (13)
H12B—C12—H12C	109.5	H21—N2—H23	107.4 (17)
N1—C13—P1	108.91 (9)	H22—N2—H23	107.3 (17)
N1—C13—H13A	109.9	P2—O2—Co1	128.45 (5)
P1—C13—H13A	109.9	Cl22—Co2—Cl24	108.699 (15)
N1—C13—H13B	109.9	Cl22—Co2—Cl23	119.352 (15)
P1—C13—H13B	109.9	Cl24—Co2—Cl23	106.345 (14)
H13A—C13—H13B	108.3	Cl22—Co2—Cl21	106.654 (14)
C13—N1—H11	109.2 (12)	Cl24—Co2—Cl21	111.513 (15)
C13—N1—H12	111.8 (12)	Cl23—Co2—Cl21	104.233 (14)
H11—N1—H12	106.4 (17)		
			
O1—P1—C13—N1	32.47 (10)	O2—P2—C23—N2	−41.65 (11)
C11—P1—C13—N1	154.27 (9)	C22—P2—C23—N2	−166.05 (9)
C12—P1—C13—N1	−90.96 (10)	C21—P2—C23—N2	80.57 (10)
C11—P1—O1—Co1	158.09 (8)	C22—P2—O2—Co1	60.81 (9)
C12—P1—O1—Co1	34.48 (10)	C21—P2—O2—Co1	−177.94 (7)
C13—P1—O1—Co1	−85.12 (9)	C23—P2—O2—Co1	−58.48 (8)

Symmetry code: (i) −*x*, −*y*, −*z*+1.

### Hydrogen-bond geometry (Å, º)

**Table:**

*D*—H···*A*	*D*—H	H···*A*	*D*···*A*	*D*—H···*A*
N1—H11···Cl1	0.867 (19)	2.437 (19)	3.1879 (12)	145.3 (16)
N1—H11···Cl24^ii^	0.867 (19)	2.730 (19)	3.2573 (13)	120.5 (15)
N1—H12···O2^i^	0.88 (2)	2.16 (2)	2.9504 (16)	150.3 (17)
N1—H13···Cl23^i^	0.87 (2)	2.38 (2)	3.2403 (12)	171.3 (17)
N2—H22···Cl1	0.920 (19)	2.250 (19)	3.1697 (13)	177.8 (15)
N2—H21···Cl21^iii^	0.924 (19)	2.288 (19)	3.2124 (12)	177.8 (16)
N2—H23···Cl22^ii^	0.86 (2)	2.71 (2)	3.3612 (13)	134.0 (16)
N2—H23···Cl24^ii^	0.86 (2)	2.70 (2)	3.2989 (12)	128.1 (16)

Symmetry codes: (i) −*x*, −*y*, −*z*+1; (ii) *x*, *y*, *z*+1; (iii) −*x*, −*y*+1, −*z*+1.
